# Knowledge, attitudes and practices of university adolescents about syphilis: a cross-sectional study in the Northeast

**DOI:** 10.11606/s1518-8787.2020054002381

**Published:** 2020-11-23

**Authors:** Rodolfo Xavier da Costa Carvalho, Telma Maria Evangelista de Araújo

**Affiliations:** I Secretaria de Saúde Municipal de Piripiri PiripiriPI Brasil Secretaria de Saúde Municipal de Piripiri. Piripiri, PI, Brasil; II Universidade Federal do Piauí Centro de Ciências da Saúde Departamento de Enfermagem TeresinaPI Brasil Universidade Federal do Piauí. Centro de Ciências da Saúde. Departamento de Enfermagem. Teresina, PI, Brasil

**Keywords:** Adolescent, Young Adult, Students, Education, Higher, Syphilis, Health Knowledge, Attitudes, Practice

## Abstract

**OBJECTIVE:**

To analyze knowledge, attitudes and practices of university adolescents about syphilis.

**METHODS:**

Cross-sectional, analytical, census-type study, developed with the universe of adolescents aged 18 and 19 years (n = 598), enrolled in three institutions of higher education in a municipality of Piauí (n = 598), which total 20 courses in the areas of Health Sciences, Applied Social Sciences, Exact and Earth, Engineering and Linguistics, Letters and Art. Data collection occurred from March to May 2019, based on a questionnaire adapted from the *Pesquisa de Conhecimentos, Atitudes e Práticas da População Brasileira* of 2013 (PCAP – Survey of Knowledge, Attitudes and Practices in the Brazilian Population), consisting of questions related to sociodemographic variables (gender, family arrangement, father's schooling, mother's schooling, skin color or race, employment, household income), knowledge, attitude and practice regarding the disease, the last three being classified by scores. The variables that presented p ≤ 0.20 in the bivariate analysis, by Pearson's chi-square test, were included in three multivariate logistic models, and the outcomes in each model were knowledge, attitude and practice, respectively; remaining at the end those at the level of p < 0.05.

**RESULTS:**

Boys have a 39.6% lower chance of having adequate/regular knowledge (OR_a_ = 0.604; 95%CI 0.415–0.878), whereas the highest chances are associated with “living alone, with relatives and friends” (OR_a_ = 4.567; 95%CI 1.417–14.719) and having a very positive/positive attitude (OR_a_ = 6.937; 95%CI 4.562–10.550). Lower chances of an adequate practice are associated with boys (OR_a_ = 0.480; 95%CI 0.301–0.766) and lower father's schooling (OR_a_ = 0.440; 95%CI 0.241–0.806).

**CONCLUSION:**

Most participants’ knowledge and attitude regarding syphilis were not sufficient to the adoption of an adequate sexual practice for the prevention of the disease, showing the need to investigate other variables that may be implicated in this cognitive incoherence.

## INTRODUCTION

Syphilis is a bacterial infection transmitted mainly during sexual intercourse and vertically from the mother to the fetus through the placenta, or even to the newborn at the time of delivery, being a substantial cause of morbidity and mortality^1^. Its overall prevalence was estimated in 0.5% (95% confidence interval [95%CI] 0.4–0.6) for men and women from 19.9 million cases, being higher in the African region, whereas America had a higher incidence of infection, with 1.7 per 1,000 women and 1.6 per 1,000 men^2^. In a national survey with Young People of the Brazilian Army between 17 and 22 years, the estimated prevalence of screened, confirmed and active infection was 1.63%, 1.09% and 0.62%, respectively^3^.

Acquired syphilis, a disease of compulsory notification since 2010, had its detection rate increased from 2.0 cases per 100,000 inhabitants to 58.1 in 2017^4^. From 2010 to 2016, the increase in the percentage of notification of acquired cases of infection in the age group of 13 to 19 years corresponded to 39.9%^5^. Thus, adolescence (between 10 and 19 years)^6^, should be recognized as a phase of higher risk of acquiring sexually transmitted infections (STI) due to its association with the development of sexual behavior^7^.

To this, we should add the concern with the adolescent during admission to the university, which is a driving factor for the expression of sexuality, because it is common in this period the awakening of the group in question to numerous and new experiences previously prohibited or limited by family proximity^8^. Contradictorily to what is believed, this context presents the predominance of people with insufficient knowledge about STI and low risk perception, allied to inconsistent condom use, sexual relations with multiple partners and use of alcohol and illicit drugs^9^.

Thus, considering the current state of syphilis and the higher risk of exposure of adolescents, especially when they are inserted in higher education and become less contemplated by the actions of primary health care (PHC) professionals because they are not included as a public of the School Health Program (SHP), the question that emerged was if this population has adequate attributes for the prevention of infection.

Therefore, our research aimed to analyze sociodemographic variables (gender, family arrangement, father's schooling, mother's schooling, skin color or race, employment, household income), knowledge, attitudes and practices of university adolescents about syphilis.

## METHODS

This is a cross-sectional, analytical, census study, developed using a knowledge, attitude and practice (KAP) survey in all three higher education institutions (HEI) of a municipality of Piauí that offer the modality of face-to-face teaching, two of which are public and one belonging to the private initiative, operating in the three shifts. Together, they offer 20 courses distributed in the areas of Health Sciences, Applied Social Sciences, Exact and Earth, Engineering and Linguistics, Letters and Arts^10^, with a total of 2,868 students enrolled.

The study population consisted of adolescents aged 18 and 19 years (n = 681). It was adopted as inclusion criterion: to be regularly enrolled in one of the selected institutions. Those that dropped out (n = 26) were excluded and there was a loss of 57 students, because they did not attend at the time of data collection despite having three returns to the HEI, resulting in 598 participants at the end. The selection of the age group mentioned was due to the assumption that a large portion of individuals would have already started sexual life.

The research instrument consisted of an adaptation of the questionnaire used in the study of the Brazilian Ministry of Health, conducted in 2013 and entitled “*Pesquisa de Conhecimentos, Atitudes e Práticas da População Brasileira*” (PCAP – Survey of Knowledge, Attitudes and Practices in the Brazilian Population)^11^ and the questionnaire applied by Souza^12^ in his *master's thesis* research with the title “*Prevalência da sífilis e fatores de risco associados em internos do sistema prisional*”.

A pilot study was conducted in February 2019, with 59 adolescents aged 18 to 19 years to improve the data collection process. The data were collected by the author of the research and by a previously trained team composed of three nursing students, from March to May 2019. Previous visits were made to the HEI to present the research and discuss the logistics of the collection, which occurred on days and times previously scheduled with students and teachers.

The team of researchers was accompanied, when possible, by the course coordinators to facilitate access to the classrooms at the time of the application of the research instruments, which were given to the participants before clarification on the content of the research and guidance on the completion, offered by the field researcher. After being answered, the participants returned it to the field researcher so that he could hold the conference according to previous guidance, respecting the ethical principles of the research.

The independent variables of the study included sociodemographic characteristics (gender, age, family arrangement, parental schooling, skin color or race, employment and family income), whereas the dependent variables were: knowledge about syphilis, attitudes towards the disease and sexual practices. To classify adolescents’ knowledge, eight questions of the instrument were analyzed. Points were added when the participant replied that: 1. Had already heard about syphilis; 2. Knew how syphilis was transmitted from one person to another; 3. Syphilis is not transmitted by contaminated water and food; 4. It cannot be transmitted by the use of public toilets; 5. It cannot be transmitted by sharing toothbrushes; 6. It can be transmitted by the non-use of condoms in sexual intercourse; 7. It can be transmitted from mother to child; 8. A lump in the genital organ is the main sign of a infection with syphilis.

Thus, based on the study by Almeida et al.^13^, knowledge was classified into three class intervals, according to the percentage of correct answers: less than 50% was considered inadequate knowledge, from 50% to 74% was considered regular knowledge and from 75% to 100% was considered adequate knowledge.

Regarding the determination of attitudes, the questionnaire was composed of six statements about syphilis, which were considered positive when the student: 1. agreed that the risk of syphilis transmission can be reduced if a person has relationships only with a faithful and uninfected partner; 2. agreed that a person with a healthy appearance may be infected; 3. agreed that condoms are the best way to prevent syphilis transmission; 4. agreed that there is a cure for syphilis; 5. agreed that the person cured of syphilis can be reinfected; 6. agreed that the pregnant woman treated properly for syphilis does not transmit the disease to her child. Subsequently, the attitude was classified according to the percentage as follows: less than 50%, negative attitude; from 50% to 74%, positive attitude; 75% to 100%, very positive attitude.

The practice of the adolescents in the study was classified, considering only those that had already started sexual activity (n = 397). Nine questions were analyzed, assigning one point to each one for the following answers: 1. used condoms in the first sexual intercourse; 2. has had no more than 10 sexual partners in his entire life; 3. has not had more than one sexual partner in the last 12 months; 4. has sexual intercourse only with a fixed partner; 5. Uses condom in all sexual relations; 6. has not had relations with more than five casual partners in the last 12 months; 7. used a condom in the last sexual intercourse; 8. had not had sexual intercourse with someone under the influence of alcohol; 9. had not had sexual intercourse with someone being under the influence of any drugs. Subsequently, scores adapted from the study by Almeida et al.^13^ were established, considering “adequate practice” when all questions were answered affirmatively; thus, from 0% to 99% was classified inadequate practice and 100% was considered adequate practice.

The data were entered in a database edited in Microsoft Office Excel 2016 and exported to the Statistical Package for the Social Sciences software (SPSS), version 21.0. Descriptive analysis of variables (sociodemographic, knowledge, attitude and practice) consisted of distributions of frequencies and percentages. For the application of inferential statistics, the variables family arrangement, father's schooling, mother's schooling, skin color or race and family income, knowledge and attitude were regrouped.

Pearson's chi-square test was used in the bivariate analysis to verify isolated associations among the variables. Those with p < 0.20 were included for the multivariate analysis of forward stepwise logistic regression^14^, with an estimate of the crude and adjusted odds ratio, in addition to a 95% confidence interval (95%CI), and three models were adjusted. The first considered sociodemographic variables and attitude as independent variables, and knowledge as a dependent variable. In the second model, attitude assumed the role of dependent variable, whereas sociodemographic variables and knowledge were the independent variables. The third model analyzed only the number of students with a sexual life initiated, that is, the variable chosen as the outcome was practice, whereas the sociodemographic variables, knowledge and attitude were arranged as independent variables. In each model, the variables at the level of p < 0.05 remained at the end.

The research was approved by the Research Ethics Committee of the Universidade Federal do Piauí under Opinion No. 3,131,024, in accordance with Resolution No. 466/2012. The participation of all adolescents in the research was expressed by signing an informed consent form, and a copy of the document was given to each participant.

## RESULTS

There was a predominance of female adolescents (56.4%), aged 19 years (54%) and who claimed to live with their father, mother and siblings (63%). The education of most parents was compatible with complete middle school (54.8%), as well as that of mothers (37.6%). The yellow or indigenous color/race prevailed, being self-declared by 66.7% of the participants. It was observed that 81.1% of the participants were unemployed, and a family income greater than one minimum wage was reported by 70.8% (Table 1).

**Table 1:** Sociodemographic characterization of the study participants. Piripiri, state of Piauí, Brazil.

Variables		n	%
Gender			
	Male	261	43.6
	Female	337	56.4
Age			
	18	275	46
	19	323	54
Family arrangement			
	Alone, with relatives or friends	96	16.1
	Father, mother and siblings	377	63.0
	Father or mother and siblings(s)	111	18.6
	With partner	14	2.3
Father's schooling			
	No schooling	38	6.4
	Middle schoola	326	54.8
	High schoola	136	22.8
	Higher educationa	95	16.0
Mother's schooling			
	No schooling	18	3.0
	Middle schoola	225	37.6
	High schoola	171	28.6
	Higher educationa	184	30.8
Skin color or race			
	White	133	22.2
	Black or brown	66	11.0
	Asian or indigenous	399	66.7
Employment			
	Yes	113	18.9
	No	484	81.1
Family income			
	Up to 1 minimum wageb	107	29.2
	More than 1 minimum wageb	259	70.8

a Complete or incomplete.

b Minimum wage: R$ 998.00.

Most adolescents had adequate/regular knowledge (64.7%) and very positive/positive attitude towards syphilis (75.4%). However, 73% of the participants that had already started sexual activity demonstrated inadequate practice for the prevention of the disease (Table 2).

**Table 2:** Classification of knowledge, attitude and practice of the adolescents in the study. Piripiri, state of Piauí, Brazil, 2019 (n = 598).

Variables		n	%
Knowledge			
	Adequate/regular	387	64.7
	Inadequate	211	35.3
Attitude			
	Very positive/positive	451	75.4
	Negative	147	24.6
Practice (n = 397)			
	Adequate	107	27.0
	Inadequate	290	73.0

The logistic regression model (Table 3) detected that boys have a 39.6% lower chance of having adequate/regular knowledge about the disease than girls, “living alone with relatives or friends” is 4.57 times more likely to have adequate or regular knowledge than “living with a partner” and the very positive or positive attitude increases by 6.94 times the chance of the adolescent having adequate/regular knowledge.

**Table 3:** Logistic models of knowledge about syphilis of adolescents with sociodemographic data and attitude. Piripiri, state of Piauí, Brazil, 2019 (n = 598).

		Knowledge		OR_b_ (95%CI)	p	OR_a_ (95%CI)	p
		Adequate/regular	Inadequate				
		n (%)	n (%)				
Gender							
	Male	150 (57.5%)	111 (42.5%)	0.570 (0.406–0.800)	**0.001**	0.604 (0.415–0.878)	**0.008**
	Female	237 (70.3%)	100 (29.7%)	1		1	
Age							
	18	180 (65.5%)	95 (34.5%)	1.062 (0.758–1.487)	0.727		
	19	207 (64.1%)	116 (35.9%)	1			
Family arrangement							
	Alone, with relatives or friends	72 (75.0%)	24 (25.0%)	4.000 (1.260–12.695)	**0.019**	4.567 (1.417–14.719)	**0.011**
	Father, mother and siblings	244 (64.7%)	133 (35.3%)	2.446 (0.831–7.199)	0.104	2.612 (0.877–7.783)	0.085
	Father or mother and sibling(s)	65 (58.6%)	46 (41.4%)	1.884 (0.612–5.797)	0.269	1.958 (0.629–6.099)	0.246
	With partner	6 (42.9%)	8 (57.1%)	1		1	
Father's schooling							
	No schooling	23 (60.5%)	15 (39.5%)	0.779 (0.358–1.694)	0.529		
	Middle school^a^	215 (66.0%)	111 (34.0%)	0.984 (0.607–1.5950)	0.947		
	High school^a^	85 (62.5%)	51 (37.5%)	0.847 (0.489–1.466)	0.552		
	Higher education^a^	63 (66.3%)	32 (33.7%)	1			
Mother's schooling							
	No schooling	13 (72.2%)	5 (27.8%)	1.197 (0.408–3.515)	0.744		
	Middle school^a^	153 (68.0%)	72 (32.0%)	0.978 (0.644–1.487)	0.918		
	High school^a^	95 (55.6%)	76 (44.4%)	0.575 (0.373–0.887)	**0.012**		
	Higher education^a^	126 (68.5%)	58 (31.5%)	1			
Skin color or race							
	White	89 (66.9%)	44 (33.1%)	1.058 (0.698–1.604)	0.792		
	Black or brown	36 (54.5%)	30 (45.5%)	0.627 (0.371–1.063)	0.083		
	Asian or indigenous	262 (65.7%)	137 (34.3%)	1			
Employment							
	Yes	74 (65.5%)	39 (34.5%)	1.037 (0.674–1.594)	0.870		
	No	313 (64.7%)	171 (35.3%)	1			
Household income							
	Up to 1 minimum wage^b^	65 (60.7%)	42 (39.3%)	0.643 (0.401–1.030)	0.065		
	More than 1 minimum wage^b^	183 (70.7%)	76 (29.3%)	1			
Attitude							
	Very positive/positive	342 (75.8%)	109 (24.2%)	7.112 (4.712–10.735)	**< 0.01**	6.937 (4.562–10.550)	**0.001**
	Negative	45 (30.6%)	102 (75.8%)	1		1	

OR_b_: gross odds ratio; OR_a_: adjusted odds ratio; 95%CI: 95% confidence interval.

a Complete or incomplete.

b Minimum wage: R$ 998.00

Values with statistical significance are presented in bold.

In the bivariate analysis, a statistically significant association of attitude with the gender variable was observed, so that male university adolescents had a 39.6% lower chance of having a very positive or positive attitude towards syphilis than female adolescents (Table 4).

**Table 4:** Association of attitude with sociodemographic aspects and knowledge of adolescents of syphilis. Piripiri, state of Piauí, Brazil.

		Attitude		OR_b_ (95%CI)	p
		Very positive/positive	Negative		
		n (%)	n (%)		
Gender					
	Male	183 (70.1%)	78 (29.9%)	0.604 (0.415–0.878)	**0.008**
	Female	268 (79.5%)	69 (20.5%)	1	
Age					
	18	212 (77.1%)	63 (22.9%)	1.183 (0.813–1.721)	0.381
	19	239 (74.0%)	84 (26.0%)	1	
Family arrangement					
	Alone, with relatives or friends	77 (80.2%)	19 (19.8%)	1.621 (0.458–5.735)	0.454
	Father, mother and siblings	280 (74.3%)	97 (25.7%)	1.155 (0.354–3.766)	0.812
	Father or mother and siblings(s)	84 (75.7%)	27 (24.3%)	1.244 (0.361–4.292)	0.729
	With partner	10 (71.4%)	4 (28.6%)	1	
Father's schooling					
	No schooling	25 (65.8%)	13 (34.2%)	0.804 (0.360–1.793)	0.593
	Middle school^a^	259 (79.4%)	67 (20.6%)	1.616 (0.964–2.708)	0.069
	High school^a^	99 (72.8%)	37 (27.2%)	1.118 (0.626–1.998)	0.706
	Higher education^a^	67 (70.5%)	28 (29.5%)	1	
Mother's schooling					
	No schooling	14 (77.8%)	4 (22.2%)	1.133 (0.355–3.618)	0.833
	Middle school^a^	175 (77.8%)	50 (22.2%)	1.133 (0.715–1.795)	0.595
	High school^a^	123 (71.9%)	48 (28.1%)	0.830 (0.517–1.332)	0.439
	Higher education^a^	139 (75.5%)	45 (24.5%)	1	
Skin color or race					
	White	103 (77.4%)	30 (22.6%)	1.088 (0.682–1.735)	0.724
	Black or brown	45 (68.2%)	21 (31.8%)	0.679 (0.385–1.196)	0.180
	Asian or indigenous	303 (75.9%)	96 (24.1%)	1	
Employment					
	Yes	91 (80.5%)	22 (19.5%)	1.440 (0.866–2.394)	0.158
	No	359 (74.2%)	125 (25.8%)	1	
Household income					
	Up to 1 minimum wage^b^	78 (72.9%)	29 (27.1%)	0.628 (0.370–1.064)	0.082
	More than 1 minimum wage^b^	210 (81.1%)	49 (18.9%)	1	

OR_b_: gross odds ratio; OR_a_: adjusted odds ratio; 95%CI: 95% confidence interval.

a Complete or incomplete.

b Minimum wage: R$ 998.00.

Note: The association with gender was not maintained in the multivariate analysis.

Values with statistical significance are presented in bold.

In the multivariate model, the variables that explained the appropriate practice were sex and father's schooling (Table 5): male university adolescents have a 52% lower chance of adequate sexual practice than those of the female, and the chance that adolescents children of parents with at most middle school education (complete or incomplete) have adequate sexual practice is 56% lower than those of parents with higher education (complete or incomplete).

**Table 5:** Multivariate analysis of the practice of adolescents with active sexual life of the study with sociodemographic aspects, knowledge and attitude. Piripiri, state of Piauí, Brazil, 2019 (n = 397).

		Practice		OR_b_ (95%CI)		OR_a_ (95%CI)	p
		Adequate	Inadequate				
		n (%)	n (%)				
Gender							
	Male	38 (20.2%)	150 (79.8%)	0.514 (0.325–0.813)	**0.004**	0.480 (0.301–0.766)	**0.002**
	Female	69 (33.0%)	140 (67%)	1		1	
Age							
	18	51 (29.5%)	122 (70.5%)	1.254 (0.803–1.958)	0.319		
	19	56 (25.0%)	168 (75.0%)	1			
Family arrangement							
	Alone, with relatives or friends	10 (14.9%)	57 (85.1%)	2.281 (0.268–19.425)	0.451		
	Father, mother and siblings	75 (31.9%0	160 (68.1%)	6.094 (0.783–47.448)	0.084		
	Father or mother and siblings(s)	21 (25.9%)	60 (74.1%)	4.550 (0.561–36.926)	0.156		
	With partner	1 (7.1%)	13 (92.9%)	1			
Father's schooling							
	No schooling	7 (29.2%)	17 (70.8%)	0.642 (0.233–1.770)	0.392	0.634 (0.227–1.772)	0.385
	Middle school^a^	50 (23.1%)	166 (76.9%)	0.470 (0.260–0.851)	**0.013**	0.440 (0.241–0.806)	**0.008**
	High school^a^	24 (26.1%)	68 (73.9%)	0.551 (0.278–1.092)	0.088	0.550 (0.275–1.101)	0.092
	Higher education^a^	25 (39.1%)	39 (60.9%)	1		1	
Mother's schooling							
	No schooling	3 (25.0%)	9 (75.0%)	1.000 (0.255–3.929)	1.000		
	Middle school^a^	44 (30.8%)	99 (69.2%)	1.333 (0.777–2.287)	0.296		
	High school^a^	29 (24.6%)	89 (75.4%)	0.978 (0.545–1.753)	0.939		
	Higher education^a^	31 (25.0%)	93 (75.0%)	1			
Skin color or race							
	White	26 (28.3%)	66 (71.7%)	0.978 (0.578–1.657)	0.935		
	Black or brown	7 (17.9%)	32 (82.1%)	1.762 (0.745–4.166)	0.197		
	Asian or indigenous	74 (27.8%)	192 (72.2%)	1			
Employment						
	Yes	18 (20.9%)	68 (79.1%)	0.657 (0.370–1.168)	0.151		
	No		221 (71.3%)	1			
Household income		89 (28.7%)					
	Up to 1 minimum wage^b^	19 (31.7%)	41 (68.3%)	1.466 (0.771–2.788)	0.242		
	More than 1 minimum wage^b^	43 (24%)	136 (76%)	1			
Knowledge							
	Adequate/regular	70 (27.0%)	189 (73.0%)	1.011 (0.634–1.611)	0.963		
	Inadequate	37 (26.8%)	101 (73.2%)	1			
Attitude							
	Very positive/positive	81 (26.0%)	230 (74.0%)	0.813 (0.481–1.374)	0.439		
	Negative	26 (30.2%)	60 (69.8%)	1			

OR_b_: gross odds ratio; OR_a_: adjusted odds ratio; 95%CI: 95% confidence interval.

a Complete or incomplete.

b Minimum wage: R$ 998.00.

Note: The association with gender was not maintained in the multivariate analysis.

Values with statistical significance are presented in bold.

## DISCUSSION

Most of the sociodemographic variables investigated are in consistence with the *V Pesquisa Nacional de Perfil Socioeconômico e Cultural dos(as) Graduandos(as) das Instituições Federais de Ensino Superior* (Ifes) (V National Survey of Socioeconomic and Cultural Profile of Undergraduate Students of Federal Institutions of Higher Education)^15^, 2018, except for the variable skin color/race. According to the research, white and brown populations are predominant in higher education, with 43.3% and 39.2%, respectively. Therefore, we believe participants have declared themselves to be yellow, when, actually, they were brown.

Adequate/regular knowledge and a very positive/positive attitude about syphilis predominated among the participants. In some studies, syphilis is referred as one of the best known STI among university students^8^^,^^9^^,^^16^^,^^17^. On the other hand, the results found contradict another Brazilian study that detected an unsatisfactory degree of knowledge about the infection among nursing university students, especially regarding prevention of transmission (27.2%) and cure (36.6%)^18^.

Despite the better result compared to this study, we must consider the proportion of adequate/regular knowledge of our study (64.7%) as low to a public in higher education, from which greater knowledge of information on the subject is expected.

In our study, the difference in knowledge about syphilis was associated with the sex of adolescents, being lower among men, a phenomenon observed in another Brazilian study, in which female adolescents had 9% more knowledge about STI than males^16^. A study conducted in Malaysia also detected a higher level of knowledge on the subject in 53% of the women surveyed, with a significant difference compared to men (44.3%)^17^.

This evidence may be related to the lower demand of men for health actions, a situation that reflects the culture of man as a strong being and incapable of getting sick^19^. Thus, the greater presence of women in health services may contribute to greater acquisition of information on syphilis, a theme commonly addressed in primary care, especially in the gestational period, due to the priority need to prevent infection in its congenital form.

The association between knowledge and family arrangement revealed that living with a partner is a predictor of less knowledge about syphilis. Young people married or living with a partner have greater individual vulnerability than single people due to the lower adoption of preventive measures and the search for information on STI/AIDS^20^.

In this sense, the fact that syphilis is not an object of interest of married participants or of participants in a stable union may represent the belief of this disease as a distant problem and, therefore, unlikely to affect people in this situation. In fact, they constitute a group susceptible to infection, as observed in a study developed in Brazil, in which the lowest use of condoms occurred among people engaged in stable relationships^21^.

This situation may occur due to the peculiarities of these relationships regarding negotiation of protection practices, which is still considered a negative social representation^22^. This conception also promotes the underestimation of the existing risk and justifies unprotected sex^21^.

Although an association between attitude and gender and knowledge was found only in the bivariate model, a similar result was observed in a study on HIV/AIDS with adolescents from Colombia, in which girls presented more favorable attitudes to negotiate condom use and avoid risky sexual behaviors than boys^23^.

The practices of adolescents that had already initiated sexual activity were predominantly inadequate, corroborating the result of a study in Rio Grande do Sul, whose prevalence of condom use among university students in the last sexual intercourse was 41.5%, considered low^22^.

This finding is worrisome, since the higher rate of inadequate sexual practice exposes adolescents to a situation of risk of contracting not only syphilis, but also other STI, and may explain the sharp growth of infection in this group in recent years^5^.

Among the participants that had already started sexual activity, inadequate practice was associated with being a boy, exposing them to a higher risk. Men have a greater variety of sexual experiences, initiate sexual activity early and have more sexual partners in life. Moreover, Men also have a higher frequency of sexual intercourse associated with the consumption of alcohol and other drugs^25^.

Despite the indications that there is a greater male involvement in risk practices, other findings contradict this position by attributing the lower use of condoms to women, such as a study conducted in Portugal, in which men more frequently mentioned the regular use of condoms^25^. In a study conducted in Rio Grande, state of Rio Grande do Sul, men also used condoms more in the last relationship than women^22^.

Despite the fact that adolescents are also involved in inadequate sexual practices, such as the non-persistent use of condoms, it can generally be considered that they tend to avoid various risky sexual behaviors, although they may have difficulties, according to the context or situation, in adopting a protection method. This often depends on your negotiation ability, which may be limited by social, economic, cultural and emotional factors^26^.

The association of the father's low schooling with inadequate sexual practices of adolescents may express the scarcity of dialogue about sex in family, especially of children with a father figure. Parents assume this role in 20.1% of cases, being the mother the main source of emotional support when they need to talk about personal problems, whereas the father assumes the sixth position, with 6.5%, after friends, partners and siblings^20^.

However, it should be noted that having them as a source of sexual education is associated with higher degrees of KAP^20^. Parents’ low schooling significantly increases early sexual activity due to limited knowledge to address sexuality issues with their children, believing that it would encourage them to have sex^27^.

A study conducted in Romania observed that girls and boys without parents’ sex education showed almost half the probability of a healthy sexual initiation^28^. Thus, it is understood that father's higher schooling constitutes a powerful factor for promoting sexual health, when intervening mainly at the time of sexual initiation of the child, whose early occurrence can lead to the development of an inadequate sexual practice.

The results also showed that the higher degree of knowledge is associated with a higher degree of attitude. However, when considering students with a sexual life, it is observed that none of the attributes influences the practice.

Although scientifically correct knowledge favors a positive attitude and, therefore, a healthy practice, this approach is not always consistent, since there are intervening factors, such as the situational circumstances surrounding the subject. They can be related to social norms, cultural patterns and roles, among others, such as momentary pressures and the social network^29^.

In a Brazilian study conducted with university students in the health area, there was no significant difference in knowledge about STI between the group that presented and the group that did not present risky sexual practice, demonstrating that only having knowledge about these infections does not ensure protection against them^30^. Therefore, it is observed the existence of a clear dissociation between academic knowledge and self-care regarding sexual health^21^.

This situation can be favored by transformations in the sexual life of adolescents with their entry in the higher education due to the emergence of new experiences, such as living away from parents and/or with friends, greater availability for night events and parties, in addition to other situations favorable to the consumption of alcohol and other drugs and unprotected sexual practices^8^^,^^9^.

In this sense, it is possible that the sexual practice of this group is also associated with other factors, such as the typical characteristics of the adolescence phase allied to the presence of a stimulating environment of risk behaviors, in addition to the lack of actions on the part of health professionals, which may judge them unnecessary, driven by mistaken ideas about the university adolescent having sufficient knowledge and being less vulnerable to syphilis and other STI.

Based on this perspective, health services should also prioritize universities to mobilize social mobilization to face this problem, as well as other injuries that affect the young population^15^.

One of the limitations of our study was the fact that we did not to consider the course or the area of knowledge of the study participants, considering the assumption about the difference in the attributes analyzed among students in the health area and other areas.

Information biases may have occurred, since we used self-declared answers, especially regarding variables related to sexual issues, which could lead respondents to change answers due to the fear of moral judgments about their conduct. However, instruments with self-response were used to minimize them.

Moreover, the research in question has representativeness, since it intended to investigate the total population defined by the criteria of interest in the municipality. In fact, our study showed relevant findings, confirmed by the scarcity of specific studies on syphilis involving this public, besides reinforcing the need for greater participation of health services in the academic scenario.

## CONCLUSION

Despite the predominance of university adolescents with adequate knowledge and attitude about syphilis, this is still a disease little known or even unknown by a considerable portion of the academic environment, which is also at risk for acquiring the infection, given the high percentage of inadequate sexual practice.

The KAP on syphilis differed according to the gender, and boys tend to have lower degrees of these attributes, which may be related to questions about gender relations.

Moreover, the investigated population has limitations to recognize the potential risk of relationships because it is possibly imbued with social conceptions that still contradict the ability to accept the need for protection against the disease.

It was observed that father's schooling positively influences the adoption of safe sexual practices and may increase the ability of the father figure to dialogue about the risks involved in unprotected sex.

Differently from what had been assumed, the knowledge and attitude about syphilis of participants with a sexual life started were indifferent to the practice of prevention against the disease, revealing the need for investigating other variables that may be implicated in this cognitive incoherence.
