# Supplementary material for: Knowledge, attitudes and practices of university adolescents about syphilis: a cross-sectional study in the Northeast
Source: Rev Saude Publica. 2020 Nov 23;54:120. doi: 10.11606/s1518-8787.2020054002381 (PMC7688255; doi:10.11606/s1518-8787.2020054002381)
Supplement: Translation

# Translation: Conhecimentos, atitudes e práticas de adolescentes universitários sobre sífilis: estudo transversal no Nordeste

**Authors:** Rodolfo Xavier da Costa Carvalho, Telma Maria Evangelista de Araújo

**Affiliations:** I PiripiriPI Brasil Secretaria de Saúde Municipal de Piripiri. Piripiri, PI, Brasil; II TeresinaPI Brasil Universidade Federal do Piauí. Centro de Ciências da Saúde. Departamento de Enfermagem. Teresina, PI, Brasil

**Keywords:** Adolescente, Adulto Jovem, Estudantes, Educação Superior, Sífilis, Conhecimentos, Atitudes e Prática em Saúde

## Abstract

**OBJETIVO:**

Analisar conhecimentos, atitudes e práticas de adolescentes universitários sobre a sífilis.

**MÉTODOS:**

Estudo transversal, analítico, do tipo censitário, desenvolvido com o universo de adolescentes de 18 e 19 anos (n = 598), matriculados em três instituições de ensino superior presencial em município do Piauí (n = 598), que juntas totalizam 20 cursos nas áreas de Ciências da Saúde, Sociais Aplicadas, Exatas e da Terra, Engenharias e Linguística, Letras e Arte. A coleta de dados ocorreu de março a maio de 2019, a partir de um questionário adaptado da Pesquisa de Conhecimentos, Atitudes e Práticas da População Brasileira de 2013, do Ministério da Saúde, constituído por perguntas referentes a variáveis sociodemográficas (sexo, idade, arranjo familiar, escolaridade dos pais, cor ou raça, atividade remunerada e renda familiar), conhecimento, atitude e prática em relação à doença, sendo as três últimas classificadas por escores. As variáveis que apresentaram valor de p ≤ 0,20 na análise bivariada, por meio do teste do qui-quadrado de Pearson, foram incluídas em três modelos logísticos multivariados, e os desfechos em cada modelo foram o conhecimento, a atitude e a prática, respectivamente; permanecendo ao final aquelas a nível de p < 0,05.

**RESULTADOS:**

O sexo masculino possui chance 39,6% menor de ter conhecimento adequado/regular (OR_a_ = 0,604; IC95% 0,415–0,878), enquanto as maiores chances estão associadas a “morar sozinho, com parentes e amigos” (OR_a_ = 4,567; IC95% 1,417–14,719) e a ter atitude muito positiva/positiva (OR_a_ = 6,937; IC95% 4,562–10,550). Menores chances de prática adequada estão associadas ao sexo masculino (OR_a_ = 0,480; IC95% 0,301–0,766) e à menor escolaridade paterna (OR_a_ = 0,440; IC95% 0,241–0,806).

**CONCLUSÃO:**

O conhecimento e a atitude da maioria dos participantes do estudo em relação à sífilis não foram suficientes para adoção de uma prática sexual adequada para a prevenção da doença, revelando a necessidade de se investigar outras variáveis que possam estar implicadas nesta incoerência cognitiva.

## INTRODUÇÃO

A sífilis é uma infecção bacteriana transmitida principalmente durante a relação sexual e verticalmente da mãe para o feto através da placenta, ou mesmo para o recém-nascido no momento do parto, sendo causa substancial de morbimortalidade^1^. Sua prevalência global foi estimada em 0,5% (intervalo de confiança de 95% [IC95%] 0,4–0,6) para homens e mulheres a partir de 19,9 milhões de casos, sendo maior na região africana, enquanto a América apresentou maior incidência da infecção, com 1,7 por 1.000 mulheres e 1,6 por 1.000 homens^2^. Já em uma pesquisa nacional com Jovens do Exército Brasileiro de 17 a 22 anos, a prevalência estimada da infecção rastreada, confirmada e ativa foi de 1,63%, 1,09% e 0,62%, respectivamente^3^.

Ressalta-se que a sífilis adquirida, agravo de notificação compulsória desde 2010, teve, a partir desse período, a sua taxa de detecção aumentada de 2,0 casos por 100 mil habitantes para 58,1 em 2017^4^. No período compreendido de 2010 a 2016, o incremento no percentual de notificação de casos adquiridos da infecção na faixa etária de 13 a 19 anos correspondeu a 39,9%^5^. Desse modo, deve-se reconhecer a adolescência, período compreendido entre 10 e 19 anos^6^, como uma fase de maior risco de adquirir infecções sexualmente transmissíveis (IST) por estar associada ao desenvolvimento do comportamento sexual^7^.

Acresce-se a esse fato a preocupação com o adolescente durante o ingresso na universidade, que consiste em fator impulsionante para a expressão da sexualidade, pois é comum nesse período o despertar do grupo em questão para inúmeras e novas experiências anteriormente proibidas ou limitadas pela proximidade familiar^8^. Contraditoriamente ao que se acredita, esse contexto apresenta a predominância de pessoas com conhecimento insuficiente sobre IST e baixa percepção de risco, aliados ao uso inconsistente do preservativo, relações sexuais com múltiplos parceiros e uso de álcool e drogas ilícitas^9^.

Assim, considerando o estado atual da sífilis e o maior risco de exposição dos adolescentes, especialmente quando encontram-se inseridos no ensino superior e se tornam menos contemplados pelas ações dos profissionais da atenção primária à saúde (APS) por não serem incluídos como público do Programa Saúde na Escola (PSE), emergiu o questionamento se essa população possui atributos adequados para a prevenção da infecção. Diante do exposto, esta pesquisa objetivou analisar características sociodemográficas, conhecimentos, atitudes e práticas de adolescentes universitários sobre sífilis.

## MÉTODOS

Trata-se de um estudo transversal, analítico, de caráter censitário, desenvolvido por meio de um inquérito de conhecimento, atitude e prática (CAP) em todas as três instituições de ensino superior (IES) de um município do Piauí que ofertam a modalidade de ensino presencial, sendo duas públicas e uma pertencente à iniciativa privada, com funcionamento nos três turnos. Juntas, perfazem uma oferta de 20 cursos distribuídos nas áreas de Ciências da Saúde, Sociais Aplicadas, Exatas e da Terra, Engenharias e Linguística, Letras e Artes^10^, com um total de 2.868 alunos matriculados.

A população do estudo foi constituída pelo universo de adolescentes de 18 e 19 anos (n = 681). Adotou-se como critério de inclusão estar regularmente matriculado em uma das instituições selecionadas. Foram excluídos os que efetuaram trancamento da matrícula (n = 26) e houve uma perda de 57 alunos, pois não compareceram por ocasião da coleta de dados, a despeito de terem sido realizados três retornos às IES, resultando ao final em 598 participantes. A seleção da faixa etária citada deveu-se ao pressuposto de que grande parcela dos indivíduos já teria iniciado a vida sexual.

O instrumento da pesquisa consistiu de uma adaptação do questionário utilizado no estudo do Ministério da Saúde, realizado em 2013 e intitulado Pesquisa de Conhecimentos, Atitudes e Práticas da População Brasileira (PCAP)^11^ e do questionário aplicado por Souza^12^ em sua pesquisa de dissertação de mestrado com o título “Prevalência da sífilis e fatores de risco associados em internos do sistema prisional”.

Realizou-se um estudo-piloto em fevereiro de 2019, com 59 adolescentes na faixa etária de 18 a 19 anos para aprimorar o processo de coleta dos dados. Os dados foram colhidos pelo autor da pesquisa e por uma equipe previamente treinada composta por três estudantes de enfermagem, no período de março a maio de 2019. Destaca-se que foram realizadas visitas prévias às IES, para apresentar a pesquisa e discutir a logística da coleta, a qual ocorreu em dias e horários previamente agendados com alunos e professores.

A equipe de pesquisadores era acompanhada, sempre que possível, pelos coordenadores de curso, para facilitar o acesso às salas de aula no momento da aplicação dos instrumentos de pesquisa, os quais eram entregues aos participantes ante esclarecimentos sobre o teor da pesquisa e orientações sobre o preenchimento, oferecidos pelo pesquisador de campo. Após respondidos, os participantes devolviam-no ao pesquisador de campo para que ele efetuasse a conferência de acordo com orientação prévia, respeitando-se os princípios éticos da pesquisa.

As variáveis independentes do estudo incluíram características sociodemográficas (sexo, idade, arranjo familiar, escolaridade dos pais, cor ou raça, atividade remunerada e renda familiar), enquanto as variáveis dependentes foram: conhecimentos sobre sífilis, atitudes em relação à doença e práticas sexuais. Para a classificar o conhecimento dos adolescentes, foram analisadas oito questões do instrumento. Foram somados pontos quando o participante respondeu que: 1. já tinha ouvido falar sobre sífilis; 2. sabia como a sífilis era transmitida de uma pessoa para outra; 3. a sífilis não pode ser transmitida por água e alimentos contaminados; 4. não pode ser transmitida pelo uso de banheiros públicos; 5. não pode ser transmitida por compartilhamento de escovas de dentes; 6. pode ser transmitida ao não usar preservativo nas relações sexuais; 7. pode ser transmitida da mãe para o bebê; 8. o nódulo ou caroço no órgão genital é o principal sinal característico da pessoa infectada com sífilis.

Assim, tomando-se como referência o estudo de Almeida et al.^13^, o conhecimento foi classificado em três intervalos de classe, conforme o percentual de acerto das respostas: menos de 50% foi considerado conhecimento inadequado, de 50% a 74% foi considerado conhecimento regular e de 75% a 100% foi considerado conhecimento adequado.

Quanto à determinação de atitudes, o questionário foi composto de seis declarações sobre sífilis, que foram consideradas positivas quando o aluno: 1. concordou que o risco de transmissão da sífilis pode ser reduzido se uma pessoa tiver relações somente com parceiro fiel e não infectado; 2. concordou que uma pessoa com aparência saudável pode estar infectada; 3. concordou que o preservativo é a melhor maneira de evitar a transmissão da sífilis; 4. concordou que existe cura para sífilis; 5. concordou que a pessoa curada de sífilis pode ser reinfectada; 6. concordou que a gestante tratada adequadamente para sífilis não transmite a doença para o seu filho. Posteriormente, a atitude foi classificada de acordo com o percentual da seguinte forma: menor que 50%, atitude negativa; de 50% a 74%, atitude positiva; de 75% a 100%, atitude muito positiva.

A prática dos adolescentes do estudo foi classificada, considerando-se apenas aqueles que já haviam iniciado a atividade sexual (n = 397). Foram analisadas nove questões, atribuindo-se um ponto a cada uma para as seguintes respostas: 1. usou preservativo na primeira relação sexual; 2. não teve mais do que 10 parceiros sexuais em toda a sua vida; 3. não teve mais do que um parceiro sexual nos últimos 12 meses; 4. tem relação sexual apenas com parceiro fixo; 5. usa preservativo em todas as relações sexuais; 6. não teve relações com mais de cinco parceiros casuais nos últimos 12 meses; 7. usou preservativo na última relação sexual; 8. não teve relação sexual com alguém estando sob efeito de álcool; 9. não teve relação sexual com alguém estando sob efeito de alguma droga. Posteriormente, estabeleceram-se escores adaptados do estudo de Almeida et al.^13^, considerando prática adequada quando todas as questões foram respondidas afirmativamente; assim, de 0% a 99% foi classificado prática inadequada e 100% foi considerado prática adequada.

Os dados foram digitados em banco editado no Microsoft Office Excel 2016 e exportados para o software *Statistical Package for the Social Sciences* (SPSS), versão 21.0, A análise descritiva das variáveis (sociodemográficas, conhecimento, atitude e prática) se constituiu de distribuições de frequências e percentuais. Para aplicação da estatística inferencial, as variáveis arranjo familiar, escolaridade do pai, escolaridade da mãe, cor ou raça e renda familiar, conhecimento e atitude foram reagrupadas.

Na análise bivariada, utilizou-se o teste do qui-quadrado de Pearson para verificar associações isoladas entre as variáveis. Aquelas que apresentaram p < 0,20 foram incluídas para a análise multivariada de regressão logística *forward stepwise*^14^, com estimativa da *odds ratio* bruta e ajustada, além de intervalo de 95% de confiança (IC95%), sendo ajustados três modelos. O primeiro considerou as variáveis sociodemográficas e a atitude como variáveis independentes, e o conhecimento como dependente. No segundo modelo, a atitude assumiu o papel de dependente, enquanto as independentes foram as variáveis sociodemográficas e o conhecimento. O terceiro modelo analisou apenas o quantitativo de estudantes com vida sexual iniciada, ou seja, a variável escolhida como desfecho foi a prática, enquanto as variáveis sociodemográficas, o conhecimento e a atitude foram dispostos como independentes. Em cada modelo, permaneceram ao final as variáveis a nível de p < 0,05.

A pesquisa foi aprovada pelo Comitê de Ética em Pesquisa da Universidade Federal do Piauí sob o Parecer nº 3.131.024, em conformidade com a Resolução nº 466/2012. A participação de todos os adolescentes na pesquisa foi expressa pela assinatura de um termo de consentimento livre e esclarecido, com entrega de cópia do documento a cada participante.

## RESULTADOS

Verificou-se predominância de adolescentes do sexo feminino (56,4%), com 19 anos de idade (54%) e que afirmaram morar com o pai, mãe e irmão(s) (63%). A escolaridade da maioria dos pais era compatível com ensino fundamental completo (54,8%), assim como a das mães (37,6%). A cor/raça amarela ou indígena prevaleceu, sendo autodeclarada por 66,7% dos investigados. Observou-se que 81,1% dos participantes não exerciam atividade remunerada, e a renda familiar maior que um salário mínimo foi informada por 70,8% (Tabela 1).

**Tabela 1:** Caracterização sociodemográfica dos adolescentes do estudo. Piripiri, PI, Brasil, 2019 (n = 598).

Variáveis		n	%
Sexo			
	Masculino	261	43,6
	Feminino	337	56,4
Idade			
	18	275	46
	19	323	54
Arranjo familiar			
	Sozinho, com parentes ou amigos	96	16,1
	Pai, mãe e irmão(s)	377	63,0
	Pai ou mãe e irmão(s)	111	18,6
	Com companheiro(a)	14	2,3
Escolaridade do pai			
	Sem escolaridade	38	6,4
	Fundamental^a^	326	54,8
	Médio^a^	136	22,8
	Superior^a^	95	16,0
Escolaridade da mãe			
	Sem escolaridade	18	3,0
	Fundamental^a^	225	37,6
	Médio^a^	171	28,6
	Superior^a^	184	30,8
Cor ou raça			
	Branca	133	22,2
	Preta ou parda	66	11,0
	Amarela ou indígena	399	66,7
Atividade remunerada			
	Sim	113	18,9
	Não	484	81,1
Renda familiar			
	Até 1 salário mínimo^b^	107	29,2
	Acima de 1 salário mínimo^b^	259	70,8

a Completo ou incompleto.

b Salário mínimo: R$ 998,00.

A maioria dos adolescentes apresentou conhecimento adequado/regular (64,7%) e atitude muito positiva/positiva para sífilis (75,4%). Entretanto, 73% dos participantes que já haviam iniciado atividade sexual demonstraram ter prática inadequada para a prevenção da doença (Tabela 2). O modelo de regressão logística (Tabela 3) detectou que o sexo masculino tem chance 39,6% menor de ter conhecimento adequado/regular sobre a doença do que as mulheres, “morar sozinho, com parentes ou amigos” tem 4,57 vezes mais chances de ter conhecimento adequado ou regular do que “morar com companheiro” e a atitude muito positiva ou positiva aumenta em 6,94 vezes a chance do adolescente ter conhecimento adequado/regular.

**Tabela 2:** Classificação do conhecimento, atitude e prática dos adolescentes do estudo. Piripiri, PI, Brasil, 2019 (n = 598).

Variáveis		n	%
Conhecimento			
	Adequado/regular	387	64,7
	Inadequado	211	35,3
Atitude			
	Muito positiva/positiva	451	75,4
	Negativa	147	24,6
Prática (n = 397)			
	Adequada	107	27,0
	Inadequada	290	73,0

**Tabela 3:** Modelos logísticos do conhecimento sobre sífilis dos adolescentes com os dados sociodemográficos e atitude. Piripiri, PI, Brasil, 2019 (n = 598).

		Conhecimento		OR_b_ (IC95%)	p	OR_a_ (IC95%)	p
		Adequado/regular	Inadequado				
		n (%)	n (%)				
Sexo							
	Masculino	150 (57,5%)	111 (42,5%)	0,570 (0,406–0,800)	**0,001**	0,604 (0,415–0,878)	**0,008**
	Feminino	237 (70,3%)	100 (29,7%)	1		1	
Idade							
	18	180 (65,5%)	95 (34,5%)	1,062 (0,758–1,487)	0,727		
	19	207 (64,1%)	116 (35,9%)	1			
Arranjo familiar							
	Sozinho, com parentes ou amigos	72 (75,0%)	24 (25,0%)	4,000 (1,260–12,695)	**0,019**	4,567 (1,417–14,719)	**0,011**
	Pai, mãe e irmão(s)	244 (64,7%)	133 (35,3%)	2,446 (0,831–7,199)	0,104	2,612 (0,877–7,783)	0,085
	Pai ou mãe e irmão(s)	65 (58,6%)	46 (41,4%)	1,884 (0,612–5,797)	0,269	1,958 (0,629–6,099)	0,246
	Com companheiro(a)	6 (42,9%)	8 (57,1%)	1		1	
Escolaridade do pai							
	Sem escolaridade	23 (60,5%)	15 (39,5%)	0,779 (0,358–1,694)	0,529		
	Fundamental^a^	215 (66,0%)	111 (34,0%)	0,984 (0,607–1,5950)	0,947		
	Médio^a^	85 (62,5%)	51 (37,5%)	0,847 (0,489–1,466)	0,552		
	Superior^a^	63 (66,3%)	32 (33,7%)	1			
Escolaridade da mãe							
	Sem escolaridade	13 (72,2%)	5 (27,8%)	1,197 (0,408–3,515)	0,744		
	Fundamental^a^	153 (68,0%)	72 (32,0%)	0,978 (0,644–1,487)	0,918		
	Médio^a^	95 (55,6%)	76 (44,4%)	0,575 (0,373–0,887)	**0,012**		
	Superior^a^	126 (68,5%)	58 (31,5%)	1			
Cor ou raça							
	Branca	89 (66,9%)	44 (33,1%)	1,058 (0,698–1,604)	0,792		
	Preta ou parda	36 (54,5%)	30 (45,5%)	0,627 (0,371–1,063)	0,083		
	Amarela ou indígena	262 (65,7%)	137 (34,3%)	1			
Atividade remunerada							
	Sim	74 (65,5%)	39 (34,5%)	1,037 (0,674–1,594)	0,870		
	Não	313 (64,7%)	171 (35,3%)	1			
Renda familiar							
	Até 1 salário mínimo^b^	65 (60,7%)	42 (39,3%)	0,643 (0,401–1,030)	0,065		
	Acima de 1 salário mínimo^b^	183 (70,7%)	76 (29,3%)	1			
Atitude							
	Muito positiva/positiva	342 (75,8%)	109 (24,2%)	7,112 (4,712–10,735)	**< 0,01**	6,937 (4,562–10,550)	**0,001**
	Negativa	45 (30,6%)	102 (75,8%)	1		1	

OR_b_: *odds ratio* bruta; OR_a_: *odds ratio* ajustada; IC95%: intervalo de confiança de 95%.

a Completo ou incompleto.

b Salário mínimo: R$ 998,00.

Valores com significância estatística estão apresentados em negrito.

Na análise bivariada observou-se associação estatisticamente significativa da atitude com a variável sexo, de forma que os adolescentes universitários do sexo masculino apresentaram chance 39,6% menor de ter atitude muito positiva ou positiva em relação à sífilis que os do sexo feminino (Tabela 4). No modelo multivariado, as variáveis que explicaram a prática adequada foram o sexo e a escolaridade do pai (Tabela 5): os adolescentes universitários do sexo masculino têm chance 52% menor de prática sexual adequada que os do feminino, e a chance de os adolescentes filhos de pais com no máximo ensino fundamental (completo ou incompleto) terem prática sexual adequada é 56% menor do que aqueles de pais com ensino superior (completo ou incompleto).

**Tabela 4:** Associação da atitude com os aspectos sociodemográficos e conhecimento dos adolescentes em relação à sífilis. Piripiri, PI, Brasil, 2019 (n = 598).

		Atitude		OR_b_ (IC95%)	p
		Muito positiva/positiva	Negativa		
		n (%)	n (%)		
Sexo					
	Masculino	183 (70,1%)	78 (29,9%)	0,604 (0,415–0,878)	**0,008**
	Feminino	268 (79,5%)	69 (20,5%)	1	
Idade					
	18	212 (77,1%)	63 (22,9%)	1,183 (0,813–1,721)	0,381
	19	239 (74,0%)	84 (26,0%)	1	
Arranjo familiar					
	Sozinho, com parentes ou amigos	77 (80,2%)	19 (19,8%)	1,621 (0,458–5,735)	0,454
	Pai, mãe e irmão(s)	280 (74,3%)	97 (25,7%)	1,155 (0,354–3,766)	0,812
	Pai ou mãe e irmão(s)	84 (75,7%)	27 (24,3%)	1,244 (0,361–4,292)	0,729
	Com companheiro(a)	10 (71,4%)	4 (28,6%)	1	
Escolaridade do pai					
	Sem escolaridade	25 (65,8%)	13 (34,2%)	0,804 (0,360–1,793)	0,593
	Fundamental^a^	259 (79,4%)	67 (20,6%)	1,616 (0,964–2,708)	0,069
	Médio^a^	99 (72,8%)	37 (27,2%)	1,118 (0,626–1,998)	0,706
	Superior^a^	67 (70,5%)	28 (29,5%)	1	
Escolaridade da mãe					
	Sem escolaridade	14 (77,8%)	4 (22,2%)	1,133 (0,355–3,618)	0,833
	Fundamental^a^	175 (77,8%)	50 (22,2%)	1,133 (0,715–1,795)	0,595
	Médio^a^	123 (71,9%)	48 (28,1%)	0,830 (0,517–1,332)	0,439
	Superior^a^	139 (75,5%)	45 (24,5%)	1	
Cor ou raça					
	Branca	103 (77,4%)	30 (22,6%)	1,088 (0,682–1,735)	0,724
	Preta ou parda	45 (68,2%)	21 (31,8%)	0,679 (0,385–1,196)	0,180
	Amarela ou indígena	303 (75,9%)	96 (24,1%)	1	
Atividade remunerada					
	Sim	91 (80,5%)	22 (19,5%)	1,440 (0,866–2,394)	0,158
	Não	359 (74,2%)	125 (25,8%)	1	
Renda familiar					
	Até 1 salário mínimo^b^	78 (72,9%)	29 (27,1%)	0,628 (0,370–1,064)	0,082
	Acima de 1 salário mínimo^b^	210 (81,1%)	49 (18,9%)	1	

OR_b_: *odds ratio* bruta; OR_a_: *odds ratio* ajustada; IC95%: intervalo de confiança de 95%.

a Completo ou incompleto.

b Salário mínimo: R$ 998,00.

Nota: A associação com o sexo não se manteve na análise multivariada.

Valores com significância estatística estão apresentados em negrito.

**Tabela 5:** Análise multivariada da prática dos adolescentes com vida sexual ativa do estudo com os aspectos sociodemográficos, conhecimento e atitude. Piripiri, PI, Brasil, 2019 (n = 397).

		Prática		OR_b_ (IC95%)	p	OR_a_ (IC95%)	p
		Adequada	Inadequada				
		n (%)	n (%)				
Sexo							
	Masculino	38 (20,2%)	150 (79,8%)	0,514 (0,325–0,813)	**0,004**	0,480 (0,301–0,766)	**0,002**
	Feminino	69 (33,0%)	140 (67%)	1		1	
Idade							
	18	51 (29,5%)	122 (70,5%)	1,254 (0,803–1,958)	0,319		
	19	56 (25,0%)	168 (75,0%)	1			
Arranjo familiar							
	Sozinho, com parentes ou amigos	10 (14,9%)	57 (85,1%)	2,281 (0,268–19,425)	0,451		
	Pai, mãe e irmão(s)	75 (31,9%0	160 (68,1%)	6,094 (0,783–47,448)	0,084		
	Pai ou mãe e irmão(s)	21 (25,9%)	60 (74,1%)	4,550 (0,561–36,926)	0,156		
	Com companheiro(a)	1 (7,1%)	13 (92,9%)	1			
Escolaridade do pai							
	Sem escolaridade	7 (29,2%)	17 (70,8%)	0,642 (0,233–1,770)	0,392	0,634 (0,227–1,772)	0,385
	Fundamental^a^	50 (23,1%)	166 (76,9%)	0,470 (0,260–0,851)	**0,013**	0,440 (0,241–0,806)	**0,008**
	Médio^a^	24 (26,1%)	68 (73,9%)	0,551 (0,278–1,092)	0,088	0,550 (0,275–1,101)	0,092
	Superior^a^	25 (39,1%)	39 (60,9%)	1		1	
Escolaridade da mãe							
	Sem escolaridade	3 (25,0%)	9 (75,0%)	1,000 (0,255–3,929)	1,000		
	Fundamental^a^	44 (30,8%)	99 (69,2%)	1,333 (0,777–2,287)	0,296		
	Médio^a^	29 (24,6%)	89 (75,4%)	0,978 (0,545–1,753)	0,939		
	Superior^a^	31 (25,0%)	93 (75,0%)	1				
Cor ou raça							
	Branca	26 (28,3%)	66 (71,7%)	0,978 (0,578–1,657)	0,935		
	Preta ou parda	7 (17,9%)	32 (82,1%)	1,762 (0,745–4,166)	0,197		
	Amarela ou indígena	74 (27,8%)	192 (72,2%)	1			
Atividade remunerada							
	Sim	18 (20,9%)	68 (79,1%)	0,657 (0,370–1,168)	0,151		
	Não		221 (71,3%)	1			
Renda familiar		89 (28,7%)					
	Até 1 salário mínimo^b^	19 (31,7%)	41 (68,3%)	1,466 (0,771–2,788)	0,242		
	Acima de 1 salário mínimo^b^	43 (24%)	136 (76%)	1			
Conhecimento							
	Adequado/regular	70 (27,0%)	189 (73,0%)	1,011 (0,634–1,611)	0,963		
	Inadequado	37 (26,8%)	101 (73,2%)	1			
Atitude							
	Muito positiva/positiva	81 (26,0%)	230 (74,0%)	0,813 (0,481–1,374)	0,439		
	Negativa	26 (30,2%)	60 (69,8%)	1			

OR_b_: *odds ratio* bruta; OR_a_: *odds ratio* ajustada; IC95%: intervalo de confiança de 95%.

a Completo ou incompleto.

b Salário mínimo: R$ 998,00.

Nota: A associação com o sexo não se manteve na análise multivariada.

Valores com significância estatística estão apresentados em negrito.

## DISCUSSÃO

A maioria das variáveis sociodemográficas investigadas converge com a V Pesquisa Nacional de Perfil Socioeconômico e Cultural dos(as) Graduandos(as) das Instituições Federais de Ensino Superior (Ifes)^15^, de 2018, exceto para a variável cor/raça. Segundo a pesquisa, brancos e pardos são predominantes no ensino superior, com 43,3% e 39,2%, respectivamente. Acredita-se, portanto, na possibilidade do preenchimento equivocado da questão por participantes que se autodeclararam amarelos, e que na verdade seriam pardos.

O conhecimento adequado/regular e a atitude muito positiva/positiva sobre a sífilis predominaram entre os participantes. Em algumas pesquisas, a sífilis é referida como uma das IST mais conhecidas entre universitários^8^^,^^9^^,^^16^^,^^17^. Em contrapartida, os resultados encontrados contrariam outro estudo brasileiro que detectou grau insatisfatório de conhecimento sobre a infecção entre estudantes universitários de enfermagem, especialmente quanto à prevenção da transmissão (27,2%) e cura (36,6%)^18^. Apesar do resultado melhor que nesse estudo, ainda se deve considerar a proporção de conhecimento adequado/regular desta pesquisa (64,7%) como baixa por se referir a um público em formação superior, do qual se espera maior apropriação de informações sobre o tema.

Neste estudo, a diferença de conhecimento sobre sífilis encontrou-se associada ao sexo do adolescente, sendo menor entre os homens, fenômeno observado em outra pesquisa brasileira, na qual o sexo feminino apresentou 9% mais conhecimento sobre IST do que o sexo masculino^16^. Um estudo realizado na Malásia também detectou um maior nível de conhecimento sobre o tema em 53% das mulheres pesquisadas, com diferença significativa para os homens (44,3%)^17^.

Essa evidência pode estar relacionada à menor procura do sexo masculino por ações de saúde, situação que reflete a cultura do homem como um ser forte e incapaz de adoecer^19^. Dessa forma, a maior presença das mulheres nos serviços de saúde pode contribuir para maior aquisição de informações sobre sífilis, tema comumente abordado na atenção primária, principalmente no período gestacional, devido à necessidade prioritária de prevenção da infecção na sua forma congênita.

A associação entre conhecimento e arranjo familiar revelou que morar com companheiro é preditor de menor conhecimento sobre sífilis. Os jovens casados ou que vivem junto com parceiro apresentam maior vulnerabilidade individual do que os solteiros devido à menor adoção de medidas preventivas e busca de informação sobre IST/aids^20^. Nesse sentido, o fato de a sífilis não ser objeto de interesse dos participantes casados ou em união estável pode representar a crença dessa doença como um problema distante e, portanto, improvável de afetar pessoas nessa situação, que, na verdade, constituem um grupo suscetível à infecção, conforme observado em um estudo desenvolvido no Brasil, no qual o menor uso de preservativos ocorria entre pessoas engajadas em relacionamentos estáveis^21^. Essa situação pode ocorrer em função das peculiaridades dessas relações, no que tange à negociação de práticas de proteção, consideradas ainda uma representação social negativa^22^. Essa concepção promove ainda a subestimação do risco existente e apena justifica o sexo desprotegido^21^.

Embora encontrada associação da atitude apenas no modelo bivariado com a variável sexo e conhecimento, resultado semelhante foi observado em um estudo com adolescentes da Colômbia sobre HIV/aids, em que as meninas apresentaram atitudes mais favoráveis para negociar o uso do preservativo e evitar comportamentos sexuais de risco do que os meninos^23^.

As práticas dos adolescentes que já haviam iniciado atividade sexual foram predominantemente inadequadas, corroborando com resultado de estudo do Rio Grande do Sul, cuja prevalência de uso preservativos entre universitários na última relação sexual foi de 41,5%, considerada baixa^22^. Essa constatação é preocupante, uma vez que o maior índice de prática sexual inadequada expõe o adolescente a uma situação de risco de contrair não apenas a sífilis e pode explicar o crescimento acentuado da infecção nesse grupo nos últimos anos^5^.

Dentre os participantes que já haviam iniciado atividade sexual, a prática inadequada foi associada ao sexo masculino, expondo-o a um maior risco. Os homens detêm maior variedade de experiências sexuais, iniciam precocemente a atividade sexual e têm mais parceiros sexuais durante a vida^24^. Ademais, os homens também apresentam maior frequência de relações sexuais associadas ao consumo de álcool e outras drogas^25^.

Em que pese os indícios de que há maior envolvimento do sexo masculino em práticas de risco, outras descobertas contrariam esse posicionamento ao atribuírem o menor uso de preservativos a mulheres, como uma pesquisa realizada em Portugal, na qual os homens mencionaram mais frequentemente o uso habitual do preservativo^25^. Em pesquisa realizada em Rio Grande, no Rio Grande do Sul, os homens também usaram mais o preservativo na última relação do que as mulheres^22^.

A despeito de as adolescentes também se envolverem em práticas sexuais inadequadas, como o uso não persistente do preservativo, no geral pode-se considerar que elas tendem a evitar diversos comportamentos sexuais de risco, embora possam ter dificuldades, de acordo com o contexto ou situação, em adotar o método de proteção. Isso depende muitas vezes da sua capacidade de negociação, que pode estar limitada por fatores sociais, econômicos, culturais e emocionais^26^.

A associação da baixa escolaridade do pai com práticas sexuais inadequadas dos adolescentes pode expressar a escassez de diálogo sobre sexo no convívio familiar, principalmente dos filhos com a figura paterna. Os pais assumem esse papel em 20,1% dos casos, sendo a mãe a principal fonte de amparo dos filhos quando precisam conversar sobre problemas pessoais, enquanto o pai assume a sexta posição, com 6,5%, atrás dos amigos, dos parceiros e dos irmãos^20^.

Entretanto, ressalta-se que tê-los como fonte de educação sexual está associado a graus mais elevados de CAP^20^. A baixa escolaridade dos pais aumenta significativamente a atividade sexual precoce devido ao conhecimento limitado para abordarem questões sobre sexualidade com os filhos, acreditando que possam incentivá-los a ter relações sexuais^27^.

Estudo realizado na Romênia observou que mulheres e homens sem a educação sexual de seus pais apresentaram quase a metade da probabilidade de uma iniciação sexual saudável^28^. Desse modo, compreende-se que a maior escolaridade do pai se constitui em um potente fator de promoção da saúde sexual, ao intervir principalmente no momento de iniciação sexual do filho, cuja ocorrência precoce pode acarretar o desenvolvimento de uma prática sexual inadequada.

Os resultados também revelaram que o maior grau de conhecimento está associado a um maior grau de atitude. Entretanto, quando se consideram os alunos com vida sexual iniciada, observa-se que nenhum dos atributos influi na prática.

Embora o conhecimento cientificamente correto favoreça a atitude positiva e, por conseguinte, uma prática saudável, nem sempre esse enfoque é tão consistente, pois existem fatores intervenientes, como as circunstâncias situacionais que rodeiam o sujeito. Elas podem relacionar-se a normas sociais, padrões culturais e papéis, entre outros, como pressões momentâneas e a própria rede social^29^. Em pesquisa brasileira realizada com universitários da área da saúde, não se encontrou diferença significativa de conhecimentos sobre IST entre o grupo que apresentou e o que não apresentou prática sexual de risco, demonstrando que apenas deter o conhecimento sobre essas infecções não assegura proteção contra elas^30^. Diante disso, percebe-se a existência de clara dissociação entre o conhecimento acadêmico e o autocuidado em relação à saúde sexual^21^.

Essa situação pode ser favorecida por transformações na vida sexual do adolescente com o seu ingresso no ensino superior, devido ao surgimento de novas experiências, como morar longe dos pais e/ou com amigos, maior disponibilidade para eventos noturnos e festas, além de outras situações favoráveis ao consumo de álcool e outras drogas, além de práticas sexuais desprotegidas^8^^,^^9^. Nesse sentido, é possível que a prática sexual deste grupo esteja também associada a outros fatores, como as características típicas da fase da adolescência aliadas à presença de um ambiente estimulador de comportamentos de risco, além da carência de ações por parte dos profissionais de saúde, que podem julgá-las desnecessárias, conduzidos por ideias equivocadas sobre o adolescente universitário possuir conhecimento suficiente e ser menos vulnerável à sífilis e a outras IST. Com base nesta perspectiva, os serviços de saúde também devem priorizar as universidades para mobilização social de enfrentamento a essa problemática, bem como de outros agravos que acometem a população jovem^15^.

Uma das limitações deste estudo foi não considerar o curso ou a área de conhecimento dos participantes do estudo, tendo em vista o pressuposto sobre a diferença dos atributos analisados entre estudantes da área de saúde e de outras áreas. Também é possível que tenham ocorrido vieses de informação, em função das respostas autodeclaradas, especialmente no que diz respeito às variáveis relacionadas às questões sexuais, que poderiam levar os entrevistados a alterarem as respostas por receio de julgamentos morais sobre a sua conduta. No entanto, para minimizá-los foram utilizados instrumentos com autorresposta.

Além disso, a pesquisa em questão possui representatividade, uma vez que pretendeu investigar o total da população definida pelos critérios de interesse no município. Aliás, cabe destacar a relevância de seus achados em virtude da escassez de estudos específicos sobre sífilis envolvendo esse público, além de reforçarem a necessidade de maior participação dos serviços de saúde no cenário acadêmico.

## CONCLUSÃO

Apesar do predomínio de adolescentes universitários com conhecimento e atitude adequados sobre sífilis, essa ainda é uma doença pouco conhecida ou mesmo desconhecida por uma parcela considerável do meio acadêmico, que também se encontra em situação de risco para aquisição da infecção, haja vista o elevado percentual de prática sexual inadequada. O CAP sobre sífilis diferiu quanto ao sexo, sendo que o masculino tende a possuir menores graus desses atributos, o que pode estar relacionado às questões sobre relações de gênero.

Ademais, salienta-se que a população investigada apresenta limitações para reconhecer o potencial de risco dos relacionamentos por estar possivelmente imbuída de concepções sociais que ainda contrariam a capacidade de aceitação da necessidade de proteção contra a doença. Observou-se que a escolaridade do pai influencia positivamente na adoção de práticas sexuais seguras, podendo ampliar a capacidade de diálogo da figura paterna sobre os riscos envolvidos no sexo desprotegido.

Diferentemente do que se havia presumido, o conhecimento e a atitude sobre sífilis dos participantes com vida sexual iniciada apresentaram-se indiferentes à prática de prevenção contra a doença, revelando a necessidade de se investigar outras variáveis que possam estar implicadas nessa incoerência cognitiva.
